# Supplementary material for: Anisotropy reveals contact sliding and aging as a cause of post-seismic velocity changes
Source: Nat Commun. 2025 Aug 15;16:7587. doi: 10.1038/s41467-025-62667-0 (PMC12356918; doi:10.1038/s41467-025-62667-0)
Supplement: Supplementary file 1 — Supplementary Information [file 41467_2025_62667_MOESM1_ESM.pdf]

Anisotropy reveals contact sliding and aging as a cause of post-seismic velocity changes - Supplementary Material

Manuel Asnar, Christoph Sens-Schönfelder, Audrey Bonnelye,
Andrew Curtis, Georg Dresen, Marco Bohnhoff

Supplementary Discussion 1 - Data pre- and post-processing

1.1 Pulse source-time correction for the acoustic traces

Since our acoustics monitoring system is not synchronized with the ultrasound pulsing system, there is a voltage threshold detection system to trigger the recording, but it does not provide the precise source time as detailed below.

A given transducer i will be sending ultrasonic pulses during the experiment. The pulse is first generated externally, sent through the transducer, and then picked up by the automatic acoustic monitoring system. When the amplitude of the pulse exceeds a given detection threshold at any receiver, the system then records 512 samples before and 3584 samples after the detection time for every single transducer in our array.

To align our observations at the precise source time, we look at the trace recordings of the pulsing channel i. Taking the very first pulse recording P_0^i as a reference, we then compute the time delay $\Delta\tau_m^i$ between the m-th pulse signal of the i-th transducer P_m^i with P_0^i in the Fourier domain. We then subtract this time delay from the trace s^i, j_m, which is the m-th trace recorded by the j-th transducer after a pulse sent by the i-th transducer.

1.2 Bimodal distribution correction on η

Our initial η measurements displayed a remarkable bimodal distribution (Figure S1) that would not go away upon averaging over sensor combinations of similar geometries. In fact the bimodal spread of the data remains when stacking measurements with different pulsing transducers which fire in fast succession. We therefore conclude that it reflects a physical change in the medium.

The system in charge of enforcing the displacement control on our sample works in discrete increments, and most likely oscillates around the desired displacement in such increments. This induces discrete, high-frequency (compared to the acoustic measurement period T_η) changes in the medium that create the bimodal distribution. We therefore correct this effect by dividing the measurement points $\eta(t_i)$ between an upper and lower band (η^U and η^L, as seen in Supplementary Figure S1, top), interpolating each of these sets on the set of all measurement times t_i, and taking the average of both bands. Since both the upper and lower band are essentially the same shifted curve with additional independent noise, averaging both bands results in a final signal noise that is significantly lower than that of the difference between the two bands (compare Supplementary Figure S1, middle and bottom).

1.3 Geometrical apparent velocity change correction on η

Our experiments involve applying $\epsilon = 10^{-5}$ of uniaxial strain upon our sample during certain periods. This, in turn, leads to small amounts of deformation within the sample, and therefore changes in the traveled path length for the waves. Since we are only interested in measuring changes in relative velocity due to material changes as opposed to geometrical changes, we remove these apparent velocity changes from the uniaxial strain phases. We do so by assuming that the sample undergoes a basic Poisson effect with a Poisson coefficient $\nu = 0.25$ [Peksa et al., 2015], and then compute the associated relative change in path length $r_k = l^1_k / l^0_k$, where l^0 is the path length before deformation and l^1 after deformation, for a given sensor combination k due to the changes in relative positions between the sensors in the deformed configuration. Finally, we obtain the apparent deformation-induced relative velocity change $\eta^{App}_k = 1 - r_k$, which we then subtract from the η measurements obtained while uniaxial strain is being applied so that only the η induced by

changes in the medium are left in our observations. Incidentally, we almost always have $\eta^{App} \ll \eta$, so that this correction only has a very limited impact on the data. This effect is of constant amplitude for a given strain level and, given that our system is displacement-controlled, has no impact on the shape of the transients. However, it could in principle alter the amplitude of the classical effect, but only does so by a comparatively small amount in practice, since the largest apparent velocity change would be within the range of 10^{-3} %, compared to the 10^{-2} % of the classical effect.

Supplementary Discussion 2 - Estimation of α

The model presented in this paper is a two-component model, with β and δ varying for each sensor combination, to which we add a time-linear component α with identical amplitude for all sensor combinations that accounts for a temperature drift.

To obtain a value for the coefficient α, it is initially considered a free parameter in a three-component inversion, along with β and δ, after which we select one $\bar{\alpha}$ as the average value of these inverted α. We then take this value $\bar{\alpha}$ and fix it for all sensor combinations before further inverting for β and δ as free parameters, resulting in our two-parameter model. We invert each set of parameters (α, β, δ) for the coefficients of a transverse anisotropy law $u\cos^2(\theta) + v$. The inverted parameters along with their anisotropy law fit are depicted in Supplementary Figure **S5**, while the coefficients obtained from the anisotropy law fit are recorded in Supplementary Table **S1**.

Supplementary Table **S1** shows that the values of u and v for the classical effect, and v for the nonclassical effect, are essentially identical for both the fixed and variable α models. One difference is in the values of u for the nonclassical effect, where the value for a variable α is twice that of when α is fixed in advance. We nonetheless argue that this does not call into question the soundness of treating the α coefficient like a fixed temperature correction. First, there is no argument in favor of treating the temperature effects as being anisotropic, and the spread of the computed values of α support that conclusion. Second, small changes in the value of α will have a disproportionate influence on the value of δ for the corresponding sensor combination, as the inverted value of δ is mostly determined by the η measurements right after a stress change, i.e. when the slope is highest, and therefore the tradeoff between α and δ is most important. For all these reasons, we conclude

that using the fixed α model as a temperature correction is a robust approach. Moreover, it does not alter our conclusions, since the values of u are still significantly different between the classical and nonclassical effects in both scenarios, hinting towards different mechanisms from which the respective effects originate.

Supplementary Discussion 3 - Uncertainty quantification on the anisotropy fits

Carrying out the fitting procedure for a given sensor combination k yields optimums β^k and δ^k. We compute the lower (resp. upper) bound β^k_{min} (resp. β^k_{max}) of the 95 % confidence interval for β^k as follows: we first compute the residuals r_{β^k} for the optimum value β^k. We then compute the residuals $r_{\beta^k_i}$ for a test value $\beta^k_i < \beta^k$ (resp. $\beta^k_i > \beta^k$). Using an F-test with a 95 % confidence level, we then test whether $r_{\beta^k_i}$ is significantly different from the reference residual r_{β^k}. If the difference is not significant, this means that β^k_i is as good a fit for our data as β^k, and β^k_i can therefore be included in the confidence interval; however, if the residuals are significantly different, this means that β^k_i no longer fits the data equally well as β^k, and is therefore outside of the confidence interval. Finally, repeating this process allows us to find the lower (resp. upper) bound of our confidence interval for β^k, and we can then reiterate for δ^k.

Supplementary Discussion 4 - Alternative models

4.1 Fitting with a straight line instead of a cosine

The law we choose for fitting the anisotropy (or lack thereof) of our inverted coefficients is of the shape $u\cos^2(\theta) + v$, where we fit for the coefficients u and v. This choice is made in line with previously known shapes for load-induced anisotropy in rocks (see Supplementary Discussion 5, along with the fact that angles rarely come into play in physical equations directly and the symmetry of our experimental setup imposes a certain periodicity with regard to θ. We could however assume no previous knowledge of the expected anisotropic behavior and simply fit our coefficients β and δ

to a linear function of θ directly, as in

$$\eta = u\left(1 - \frac{\theta}{\theta_{max}}\right) + v \tag{1}$$

where we once again invert for u and v. We normalize by $\theta_{max} = 90°$ and swap the sign in order to have u and v values that can directly be compared to those in the cosine-square anisotropy law. Doing so for our dataset yields the results shown in Supplementary Figure **S4** and the values reported in Supplementary Table **S1**. We notice that the values for u and v, as well as those for their standard errors, are strikingly similar, which should not be surprising given that our cosine-squared law was inverted using values of θ over the 40°-90° range where it is straightest. Thus, the core of our argument is preserved, in that both models show that the classical and nonclassical effects exhibit fundamentally different anisotropies, with the nonclassical effect being significant along every ray path. Since our cosine-square law is most in line with our current physical understanding of the classical effect, it is the one we are going for.

4.2 Alternative 10-component model: fitting stress states individually

To challenge our 2-component model, we attempted to fit our data using a 10-component model where the classical and nonclassical effects were described with different parameters for each different stress state, on top of a freely varying linear trend parameter α. The model is as follows (see also Supplementary Figure **S6**):

$$\eta(t;\theta) = \alpha L(t) + \sum_{i=1}^{5} \beta_i(\theta) C_i(t) + \sum_{i=1}^{4} \delta_i(\theta) R_i(t) \tag{2}$$

Just like we do for our other models, we invert each set of parameters $(\alpha, \beta_1, ..., \delta_4)$ for the coefficients of a transverse anisotropy law $u\cos^2(\theta) + v$. We display the computed parameters and the anisotropy fits in Supplementary Figure **S7**, and record the obtained anisotropy fit coefficients in Supplementary Table **S1**, along with those of our model with a variable α.

The table shows for the classical component that the only significant values are obtained for β_2 and β_4, corresponding to the times when the sample is under static uniaxial strain (see Supplementary Figure **S6**), while when the sample is under hydrostatic compression only, the classical

components are virtually null. This further confirms that the classical effect is related to the stress state of the sample and is a static effect, thereby justifying the shape of the classical component as used in the main paper.

For the nonclassical effects, we note that with the exception of the value of u for δ_4, the obtained anisotropy parameters are remarkably similar for each transient, also lending strength to the assumed shape of our transient component $R(t)$ in our main 2-parameter model. The values of the intercepts v are also within the same range as the ones for our 2-parameter model, and the u coefficients, although larger than for the 2-parameter model, are of the same order of magnitude as in the 3-parameter model, and still significantly different from the u values for the classical component.

Also worth noting is the fact that the inversions for α for the 3-component and 10-component models are also remarkably similar, so that the tradeoff discussion between the nonclassical component and the linear trend component can apply to both.

Although 10 components can naturally better capture the finer variations within our data, doing so does not provide any additional insights and still supports the observation of the much simpler 2-component model, that the anisotropy of the classical and nonclassical effects are significantly different. The nonclassical effect is significant for all propagation directions and less anisotropic than the classical effect while the latter one almost vanishes in the horizontal direction. The 10-parameter inversion thus adds unnecessary complexity to the analysis and we present the 2-component model in the main text.

Supplementary Discussion 5 - Expected anisotropy of the classical effect

We can theoretically derive the anisotropic load-induced relative velocity changes in classically nonlinear elastic materials, i.e. where the nonlinearity is described using the Murnaghan coefficients [Murnaghan, 1937], using the applied stress σ, the Lamé parameters and the third-order elastic constants l, m and n. Here, $\sigma > 0$ denotes compression, which also flips the signs of l, m and n.

We define our classically-induced relative velocity change as follows:

$$\eta_C(\theta;\sigma) = \frac{V_P(\theta;\sigma) - V_{P_{ref}}}{V_{P_{ref}}} \tag{3}$$

where $\rho V_{P_{ref}}^2 = \lambda + 2\mu$ is the P-wave velocity before the load is applied, and $V_P(\theta;\sigma)$ is the load- and angle-dependent absolute P-wave velocity under the uniaxial stress. In the next steps, we omit the explicit dependence on σ for clarity. We can express $V_P(\theta)$ as a function of $V_{P_\parallel}$, which is the velocity along the principal stress direction, and $A_P(\theta)$, which is a load-dependent anisotropy coefficient, in the following way [Johnson and Rasolofosaon, 1996]:

$$V_P(\theta) = (1 - A_P(\theta))V_{P_\parallel}$$

Reinjecting these terms in Equation 3, we finally get an expression of the form

$$\eta_C(\theta) = u\cos^2(\theta) + v \tag{4}$$

where, with $K = \lambda + \frac{2}{3}\mu$ being the bulk compressibility modulus, we have

$$u = \sigma a_C\sqrt{1 + \sigma b_C} \qquad v = (1 - \sigma a_C)\sqrt{1 + \sigma b_C} - 1 \tag{5}$$

$$a_C = \frac{2\lambda + 5\mu - 2m}{2\mu(\lambda + 2\mu)} \qquad b_C = \frac{-2l + \lambda + \frac{\lambda+\mu}{\mu}(4\lambda + 10\mu - 4m)}{3K(\lambda + 2\mu)} \tag{6}$$

(7)

which gives us the final expression for the load-induced anisotropic change in relative velocity. This theoretical expression is only valid for small values of stress, which is our case here, as well as in the case of a purely uniaxial load.

This derivation matches our observations in several qualitative aspects. First, we note that, in these expressions, η_C depends on the sign of the applied strain *via* the applied stress σ, and we have $\eta_C = f(\Delta\varepsilon_n)$. Second, the dependence of η_C as a linear function of $\cos^2(\theta)$ matches the measured anisotropy of $\beta(\theta)$. Using values of l, m and n for Berea sandstone [Renaud et al., 2016], we find

values of $u = 38 \times 10^{-4}$ and $v = 4.8 \times 10^{-4}$, whose discrepancy with our inverted values are within the previously reported range in the literature [Winkler and McGowan, 2004].

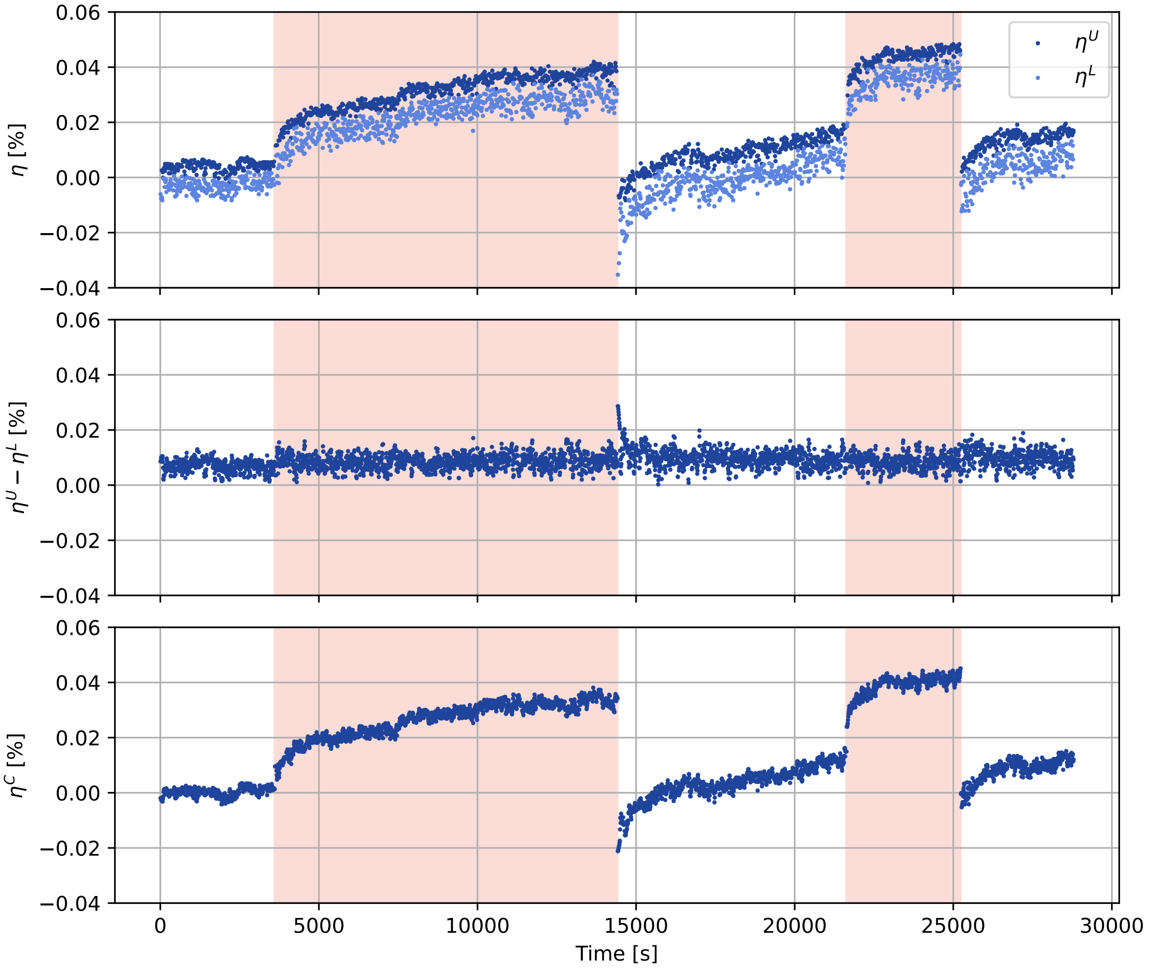

Supplementary Figure S1: **Bimodal distribution correction of the η measurements** An example of a set of uncorrected η measurements with the distinct upper and lower bands (top), the difference between the upper and lower bands (middle), and the corrected η^C obtained by averaging both bands (bottom). Note how, in the uncorrected version, both bands exhibit the same low-frequency variations (e.g. around 16 000 and 27 500), and how the width between the two bands is essentially constant throughout the whole experiment.

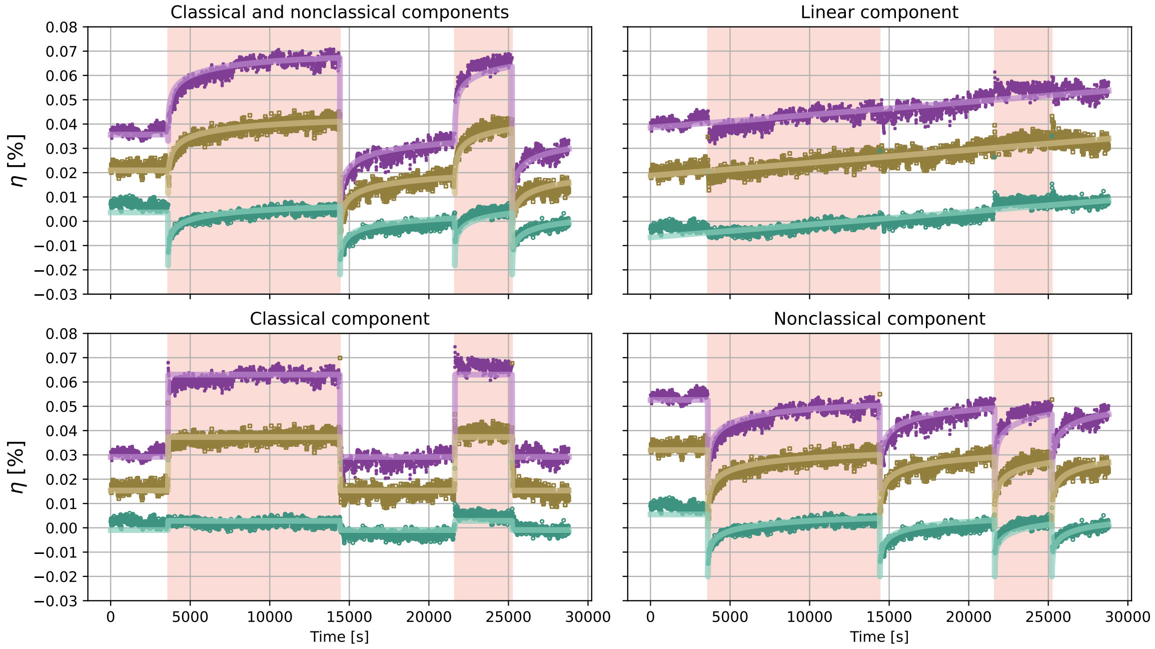

Supplementary Figure S2: **Relative velocity change measurements with different model components** Relative velocity change measurements η for three different values of θ. From top to bottom in each panel: 40°, 72° and 90°. Solid lines show the respective partial model fits, with the undesired components subtracted from the total model fit. Vertical offsets have been adjusted across panels for ease of reading.

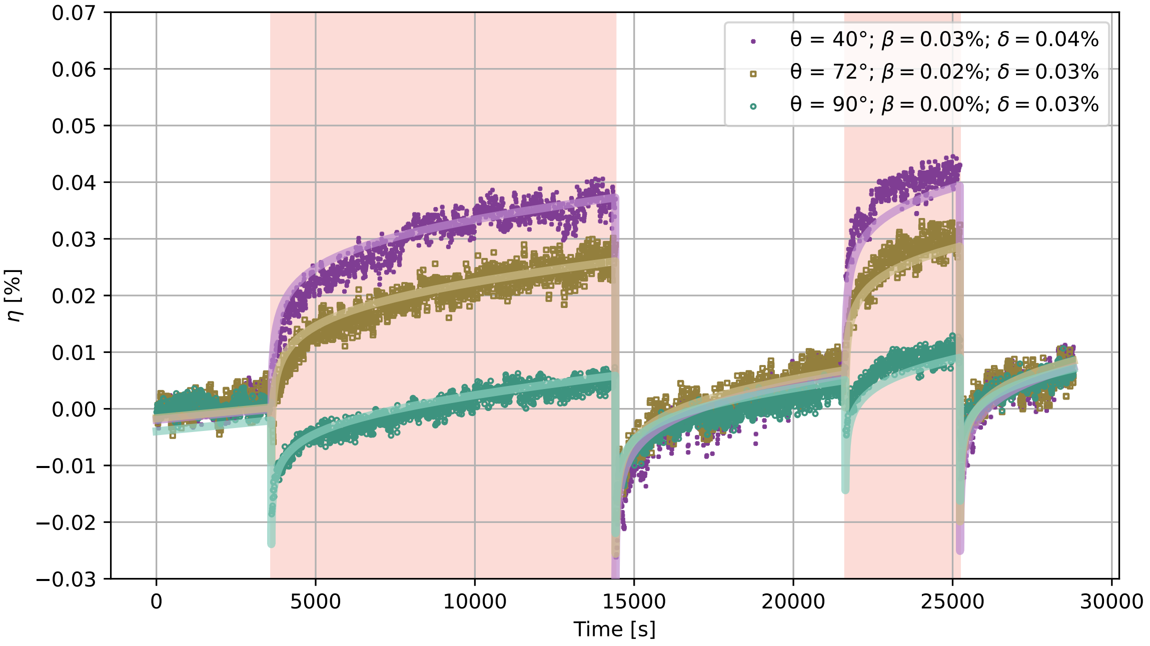

Supplementary Figure S3: **Relative velocity change measurements and model components** Relative velocity change measurements η for three different values of θ (see Figure 1 in the main manuscript). The origin of each scatter plot was aligned for easier comparison.

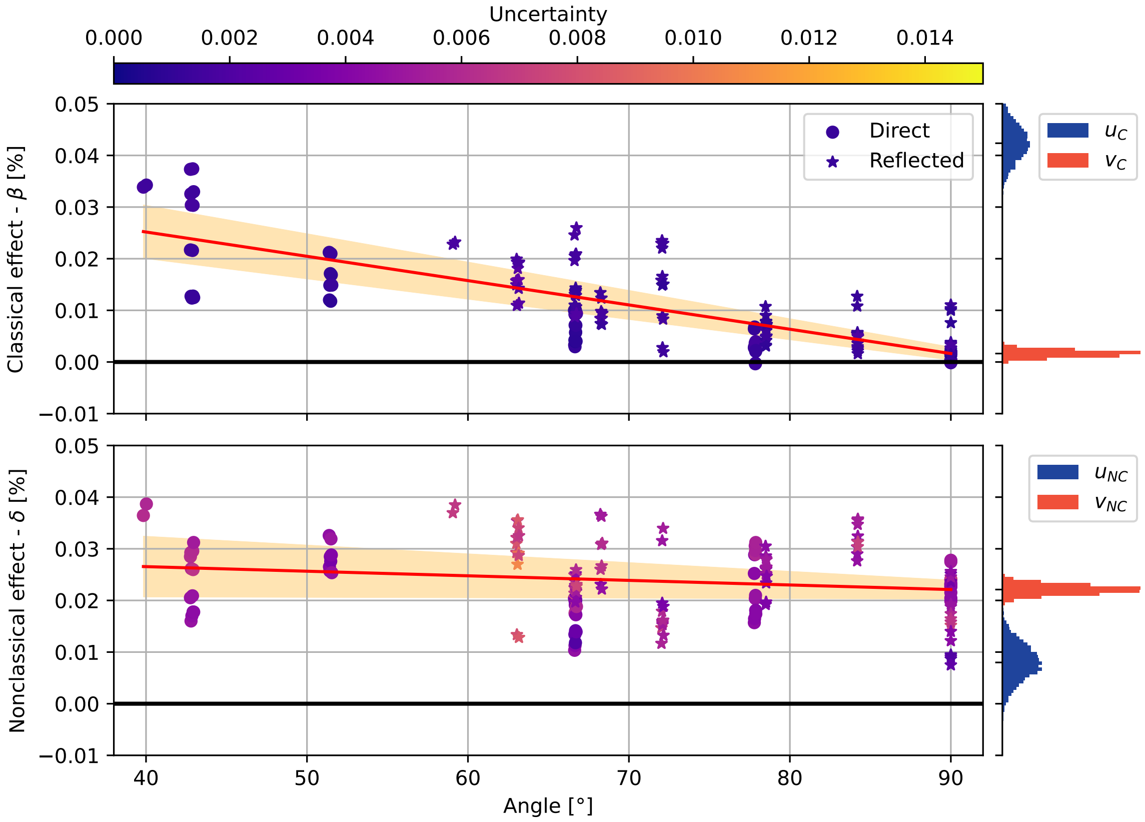

Supplementary Figure S4: **Anisotropy of the classical and nonclassical effects** Results of the fitting of β and δ parameters to a law of the form $u(1 - \theta/\theta_0) + v$. Dots indicate measurements resulting from direct P-wave arrivals; stars are for reflected P-wave arrivals. Color of symbols shows uncertainty of individual measurements. The red curve is the average model with orange bands indicating the 95 % confidence interval. The histograms show the results of the 5000-iteration bootstrap sampling inversion of the u and v coefficients.

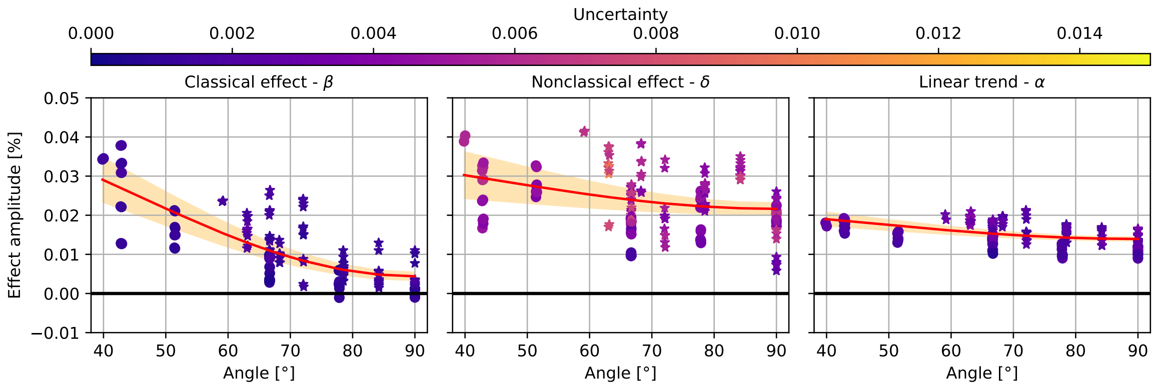

Supplementary Figure S5: **Anisotropy fit results for the 3-component model** Dots are for values obtained looking at direct P-wave arrivals, stars with reflected P-wave arrivals. See main manuscript for more details on the inversion process.

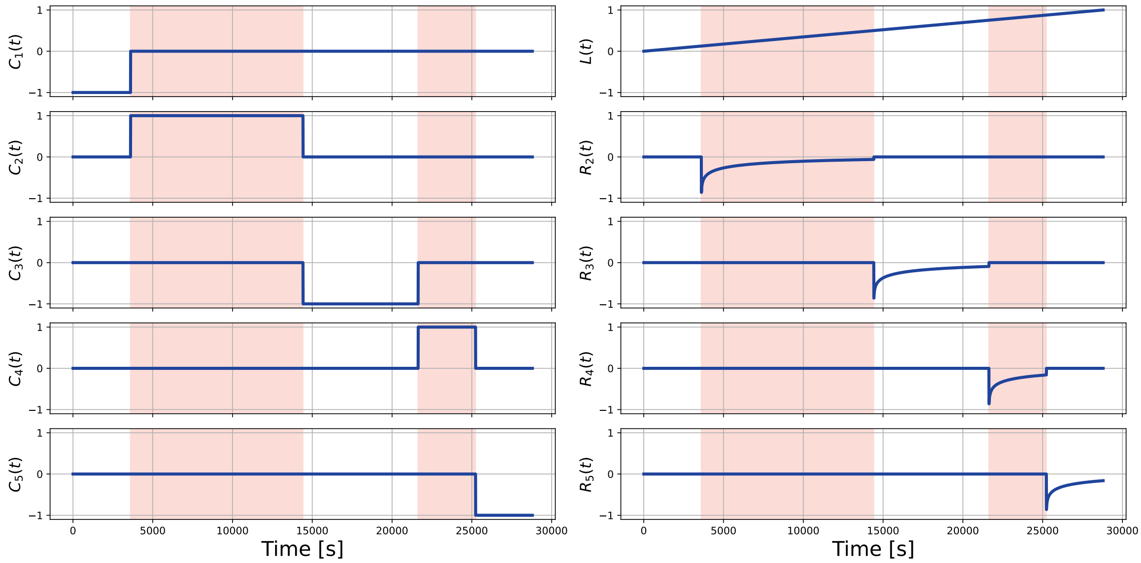

Supplementary Figure S6: **Individual components for the 10-component model** We have five individual step components for the classical effect, and four individual components for the transient effect

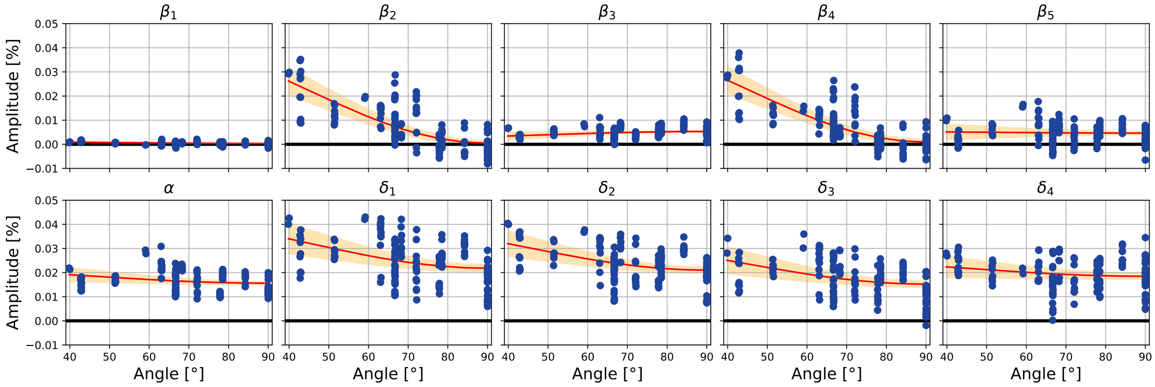

Supplementary Figure S7: **Anisotropy fit results for the 10-component model** Uncertainties on the individual parameter values were not computed for this model.

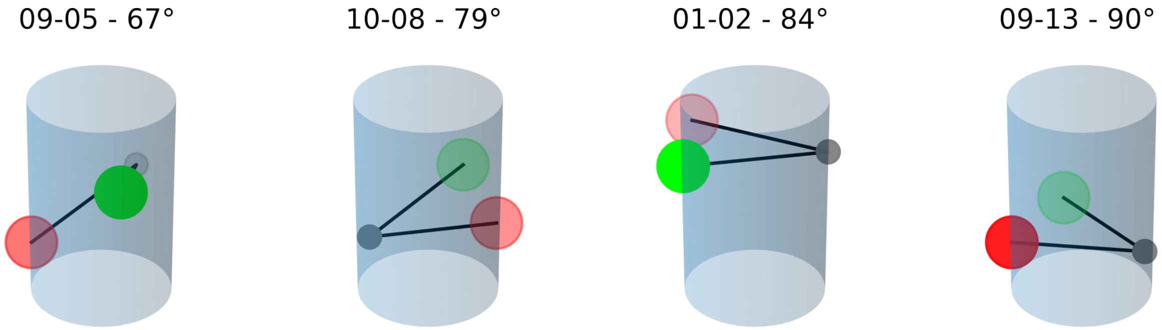

Supplementary Figure S8: **Examples of reflected path geometries** Some selected reflected ray paths that were included in our analysis for different sensor combinations, with the pulsing (green) and receiving (red) transducers and the reflection point (grey). Transducer numbers are as shown in Figure 4 of the main manuscript.

Model	Coefficients (× 10^{-2} %) u	σ_u	v	σ_v
Fixed α				
β	4.07	0.39	0.45	0.05
δ	0.72	0.35	2.27	0.07
Linear fit				
β	4.24	0.35	0.16	0.06
δ	0.80	0.36	2.21	0.09
Variable α				
α	0.86	0.11	1.39	0.03
β	4.18	0.39	0.44	0.06
δ	1.46	0.37	2.16	0.08

Model	Coefficients (× 10^{-2} %) u	σ_u	v	σ_v
Individual fits				
α	0.60	0.19	1.56	0.04
β_1	0.10	0.02	0.04	0.01
β_2	4.38	0.40	0.05	0.06
β_3	−0.32	0.10	0.53	0.03
β_4	4.36	0.39	0.09	0.06
β_5	0.09	0.21	0.46	0.04
δ_1	2.05	0.40	2.18	0.09
δ_2	1.87	0.34	2.10	0.07
δ_3	1.67	0.36	1.52	0.07
δ_4	0.66	0.27	1.85	0.07

Supplementary Table S1: **Anisotropy fit coefficients for each model** Coefficients u and v and their respective standard errors resulting from the anisotropy law fit $\gamma = u\cos^2(\theta) + v$ for each parameter γ and for our different models

References

[Johnson and Rasolofosaon, 1996] Johnson, P. A. and Rasolofosaon, P. N. J. (1996). Nonlinear elasticity and stress-induced anisotropy in rock. *Journal of Geophysical Research: Solid Earth*, 101(B2):3113–3124.

[Murnaghan, 1937] Murnaghan, F. D. (1937). Finite Deformations of an Elastic Solid. *American Journal of Mathematics*, 59(2):235.

[Peksa et al., 2015] Peksa, A. E., Wolf, K.-H. A., and Zitha, P. L. (2015). Bentheimer sandstone revisited for experimental purposes. *Marine and Petroleum Geology*, 67:701–719.

[Renaud et al., 2016] Renaud, G., Talmant, M., and Marrelec, G. (2016). Microstrain-level measurement of third-order elastic constants applying dynamic acousto-elastic testing. *Journal of Applied Physics*, 120(13):135102.

[Winkler and McGowan, 2004] Winkler, K. W. and McGowan, L. (2004). Nonlinear acoustoelastic constants of dry and saturated rocks. *Journal of Geophysical Research: Solid Earth*, 109(B10):2004JB003262.
