# Supplementary figures and images for: Anisotropy reveals contact sliding and aging as a cause of post-seismic velocity changes
Source: Nat Commun. 2025 Aug 15;16:7587. doi: 10.1038/s41467-025-62667-0 (PMC12356918; doi:10.1038/s41467-025-62667-0)

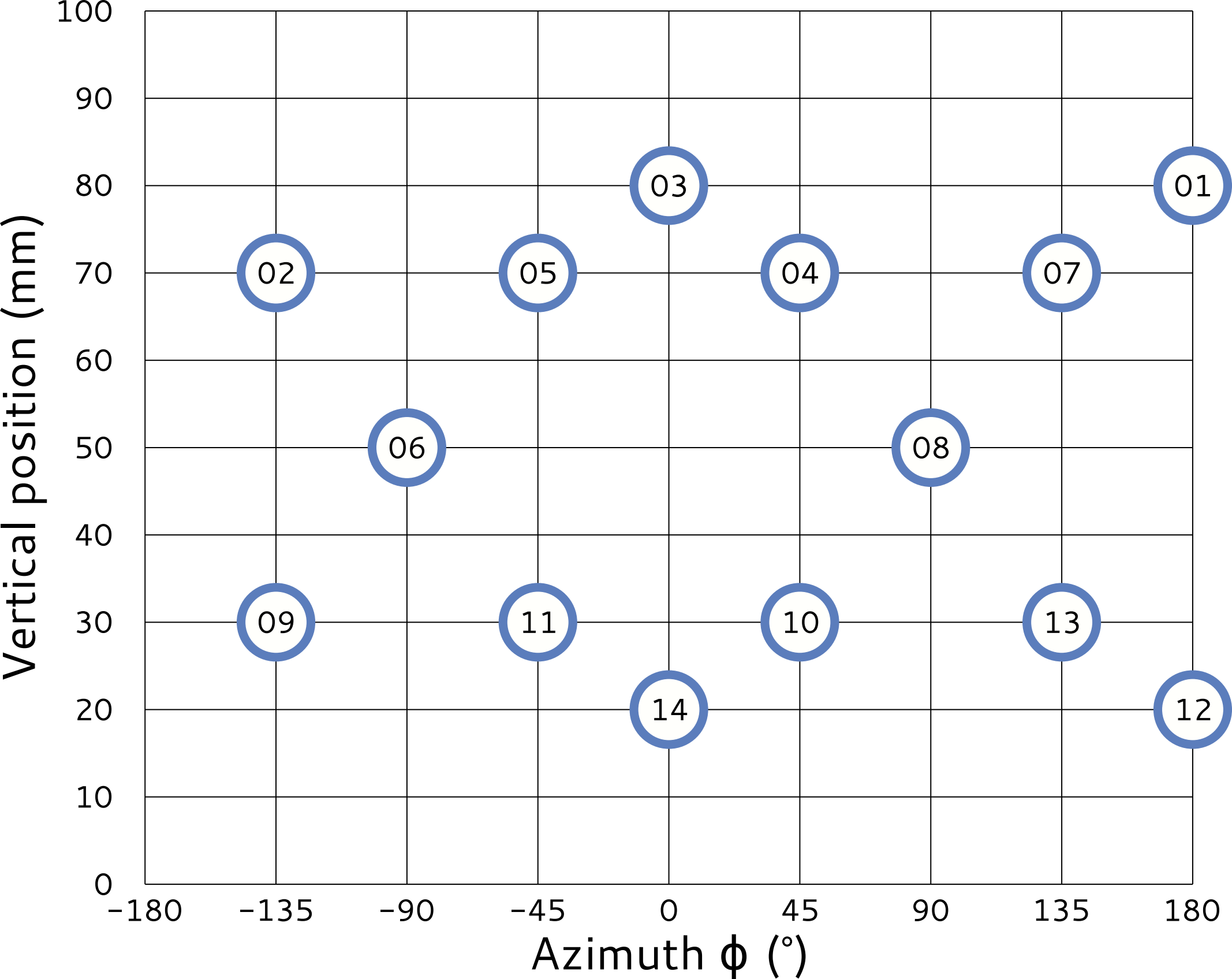

Supplement: Supplementary file 3 — Source Data [file 41467_2025_62667_MOESM3_ESM.zip › code/data/sensor_map.pdf]

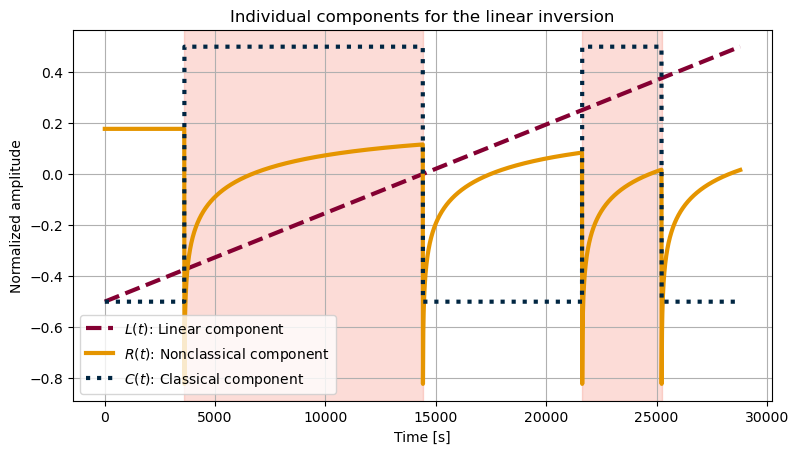

Supplement: Supplementary file 3 — Source Data [file 41467_2025_62667_MOESM3_ESM.zip › code/nb_output/model.png]

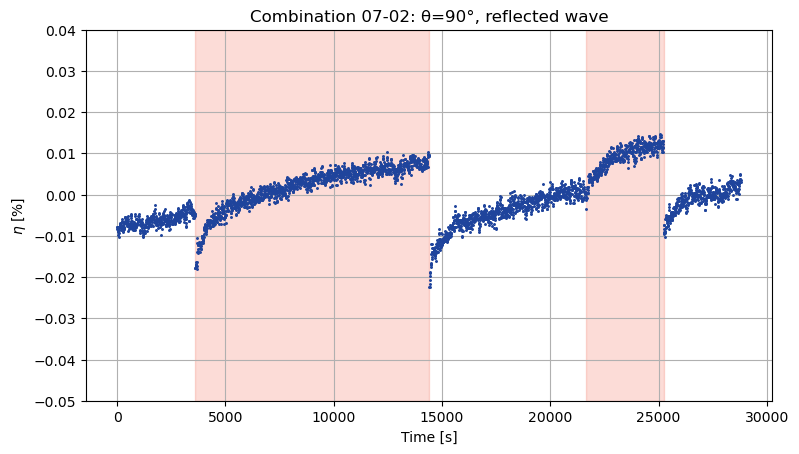

Supplement: Supplementary file 3 — Source Data [file 41467_2025_62667_MOESM3_ESM.zip › code/nb_output/eta.png]

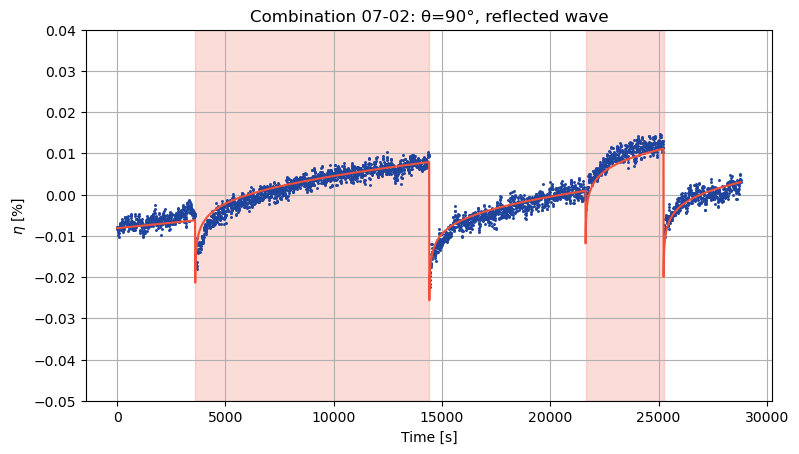

Supplement: Supplementary file 3 — Source Data [file 41467_2025_62667_MOESM3_ESM.zip › code/nb_output/eta_model.png]
